# Pharmacological Basis for the Use of Evodiamine in Alzheimer’s Disease: Antioxidation and Antiapoptosis

**DOI:** 10.3390/ijms19051527

**Published:** 2018-05-21

**Authors:** Yongfeng Zhang, Jiaqi Wang, Chunyue Wang, Zhiping Li, Xin Liu, Jun Zhang, Jiahui Lu, Di Wang

**Affiliations:** 1 School of Life Sciences, Jilin University, Changchun 130012, China; yongfeng17@mails.jlu.edu.cn (Y.Z.); wangjq1316@mails.jlu.edu.cn (J.W.); chunyue17@mails.jlu.edu.cn (C.W.); liuxin1314@mails.jlu.edu.cn (X.L.); lujh545@jlu.edu.cn (J.L.); 2 Department of Pharmacology, College of Basic Medical Sciences, Jilin University, Changchun 130006, China; zpli15@mails.jlu.edu.cn; 3 Changchun Shengjinnuo Biological Pharmaceutical Co., Ltd., Changchun 130000, China; sjnzhangjun@126.com

**Keywords:** Evodiamine, Alzheimer’s disease, apoptosis, oxidative stress, cholinergic system

## Abstract

Evodiamine (Evo), a major alkaloid compound isolated from the dry unripened fruit of Evodia fructus, has a wide range of pharmacological activities. The present study sought to explore the neuroprotective effects of Evo in l-glutamate (l-Glu)-induced apoptosis of HT22 cells, and in a d-galactose and aluminum trichloride-developed Alzheimer’s disease (AD) mouse model. Evo significantly enhanced cell viability, inhibited the accumulation of reactive oxygen species, ameliorated mitochondrial function, increased the B-cell lymphoma-2 protein content, and inhibited the high expression levels of Bax, Bad, and cleaved-caspase-3 and -8 in l-Glu-induced HT22 cells. Evo also enhanced the phosphorylation activities of protein kinase B and the mammalian target of rapamycin in the l-Glu-induced HT22 cells. In the AD mouse model, Evo reduced the aimless and chaotic movements, reduced the time spent in the central area in the open field test, and decreased the escape latency time in the Morris water maze test. Evo reduced the deposition of amyloid beta 42 (Aβ42) in the brain, and increased the serum level of Aβ42, but showed no significant effects on Aβ40. In addition, six weeks of Evo administration significantly suppressed oxidative stress by modulating the related enzyme levels. In the central cholinergic system of AD mice, Evo significantly increased the serum levels of acetylcholine and choline acetyltransferase and decreased the level of acetylcholinesterase in the serum, hypothalamus, and brain. Our results provide experimental evidence that Evo can serve as a neuroprotective candidate for the prevention and/or treatment of neurodegenerative diseases.

## 1. Introduction

Alzheimer’s disease (AD), a chronic and progressive neurodegenerative disorder generally related to ageing, is characterized by cognitive dysfunction, impaired memory, and poor prognosis [1]. Although the pathological cause of AD is still mostly unknown, past findings have proven that patients with AD show an accumulation of abnormally folded beta-amyloid (Aβ) in senile plaques and neuronal apoptosis [2]. Oxidative stress has been reported as one of the key causes of neuronal apoptosis during the development of AD, which is associated with the remarkable accumulation of reactive oxygen species (ROS), mitochondrial dysfunction, and calcium accumulation [3]. ROS are catalyzed by a combination of redox active metal ions and Aβ in AD [4]. Severe damage to the cholinergic system, including decreased choline uptake and reduced choline acetyltransferase (ChAT) activity, have been noted in AD patients; meanwhile, the damaged cholinergic system may be further responsible for Aβ plaque formation and increasing phosphorylation of tau protein [5,6,7].

Glutamate, an excitatory neurotransmitter in the brain, can cause neuron death mainly related to oxidative damage by preventing the synthesis of intracellular antioxidants and eventually causing an accumulation of ROS [8]. As an in vitro experimental model of neuronal death in neurodegenerative disease, l-glutamate (l-Glu)-induced HT22 cell (mouse hippocampal neuronal cells) apoptosis is at least partially related to the caspase-dependent mitochondrial apoptosis process [8]. Aluminum trichloride (AlCl_3_) can induce the generation of free radicals and neurotoxicity in the brain, leading to degenerative diseases [9]. Exogenous d-galactose (d-gal) induces learning and memory impairments in experimental animals related to the imbalance of anti- and pro-oxidation [9,10]. Our group has successfully confirmed the neuroprotective effects of *Amanita caesarea* and *Armillaria mellea* and improvement of AD-like behavior in AlCl_3_ plus d-gal-developed AD mice [11].

**Figure 1:**
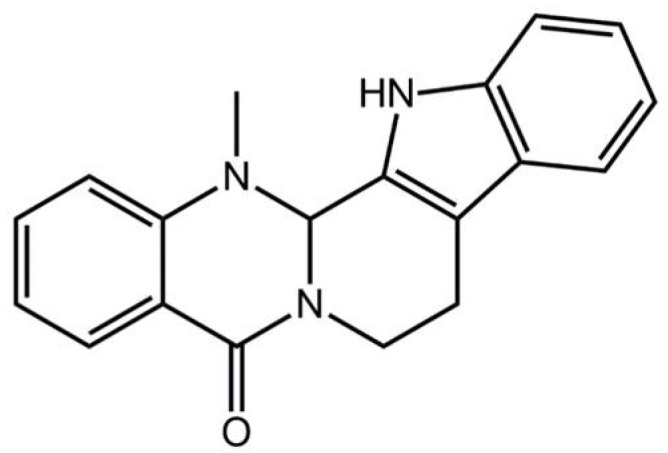
Chemical structure of Evodiamine.

Recently, great progress has been made in the use of natural compounds for improving the symptoms of AD, which is worthy of further research and development [12]. Evodiamine (Evo), a major alkaloid compound isolated from the dry unripened fruit of Evodia fructus, exhibits various pharmacological effects, such as anti-inflammatory, analgesic, immunomodulatory, and hypoglycemic activities [13]. Its structure is shown in Figure 1. Evo reduces the expression of inflammatory cytokines in senescence-accelerated mouse/prone 8 (SAMP8) and amyloid precursor protein and presenilin 1 (APP^swe^/PS1^ΔE9^) transgenic mouse models of AD, and increases the uptake of glucose in brain tissues [12,14]. Evo induces cellular apoptosis by suppressing phosphoinositide 3-kinase/protein kinase B (Akt) signaling and activating mitogen-activated protein kinase (MAPK) in glioblastoma multiforme to regulate apoptotic protein expression [15]. However, the neuroprotective effects of Evo, especially on AD symptoms, and the underlying mechanisms have not yet been systematically reported. 

In the present study, we used l-Glu-induced HT22 apoptotic cells and d-gal plus AlCl_3_-induced AD mice to investigate the neuroprotective effects of Evo. Encouragingly, Evo protected l-Glu-damaged HT22 cells by ameliorating cellular viability, reducing the proportion of apoptotic cells, restoring the dissipation of mitochondrial transmembrane potential (MMP), regulating apoptosis-related protein expression such as B-cell lymphoma-2 (Bcl-2) family members and caspase protein, and the Akt/mammalian target of the rapamycin (mTOR) signal pathway. Evo also improved the AD-like behavior and regulated the physiological and biochemical indices of AD mice. Our study provides experimental evidence that Evo could be used as a potential agent in clinical applications for the treatment of AD.

## 2. Results

### 2.1. Evo Protects HT22 Cells against l-Glu Damage via Regulation of Mitochondrial Function

**Figure 2:**
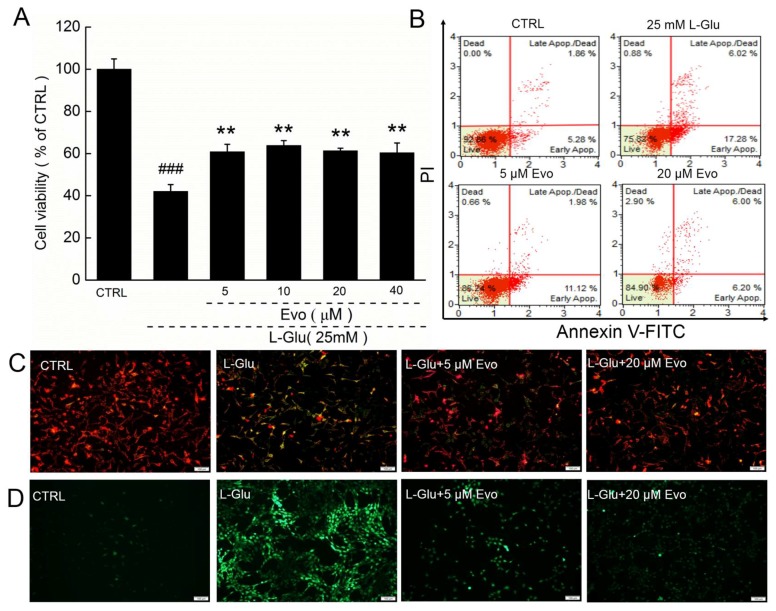
Evo showed neuroprotective effects on HT22 cells against l-Glu-caused damage. (**A**) 3-h pre-treatment with Evo enhanced cell viability in HT22 cells exposed to 25 mM l-Glu for 24 h. ### *p* < 0.001 vs. CTRL. ** *p* < 0.01 vs. l-Glu-treated cells; (**B**) 3-h pre-treatment of Evo reduced the apoptosis rate in HT22 cells exposed to l-Glu for 24 h (*n* = 10); (**C**) 3-h pre-treatment of Evo ameliorated the dissipation of MMP in HT22 cells exposed to l-Glu for 12 h (Scale bar: 100 μm; *n* = 10); (**D**) 3-h pre-treatment of Evo inhibited the over-accumulation of ROS in HT22 cells exposed to l-Glu for 12 h (Scale bar: 100 μm; *n* = 10).

The addition of 25 mM l-Glu resulted in a 58.1% reduction in cell viability in HT22 cells (*p* < 0.001; Figure 2A), which was significantly improved by 3-h pre-treatment of Evo at doses from 5 to 40 μM (*p* < 0.01; Figure 2A). In l-Glu-exposed HT22 cells, 23.3% of cells were apoptotic, compared with only 12.2% of cells that were pre-treated with 20 μM Evo (Figure 2B). 20 μM Evo alone had no significant effect on cell viability and apoptosis in HT22 cells (Figure S1A,B).

The imbalance in the MMP is an early event in mitochondrial dysfunction, and Evo strongly restored the dissipation of the MMP caused by l-Glu, as indicated by the enhanced red fluorescence intensity and reduced green fluorescence intensity (Figure 2C). The over-accumulation of ROS causes oxidative stress and finally leads to cell death. Three-hour pre-treatment with 5–20 μM Evo and co-exposure to 25 mM l-Glu strongly suppressed the l-Glu-caused over-production of intracellular ROS, as suggested by the suppressed green fluorescence intensity (Figure 2D). 20 μM Evo alone had no significant effect on MMP and ROS in HT22 cells (Figure S1C,D).

### 2.2. Evo Ameliorates the Expression Levels of Apoptosis-Related Proteins in HT22 Cells

**Figure 3:**
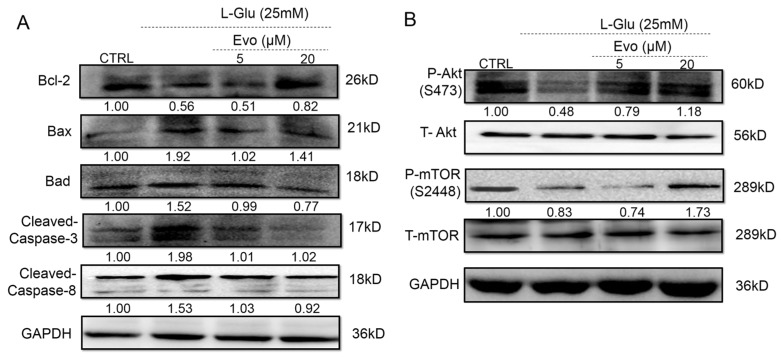
Evo regulated the expression levels of apoptosis-related proteins in HT22 cells exposed to l-Glu for 24 h. Evo increased the expression levels of Bcl-2, P-Akt, and P-mTOR, and reduced the expression levels of Bax, Bad, and cleaved-caspase-3 and -8. Quantification data were normalized by (**A**) glyceraldehyde-3-phosphate dehydrogenase (GAPDH) and (**B**) corresponding total proteins and the average fold increase in band intensity compared with the CTRL group are marked, respectively (*n* = 6).

The Bcl-2 family proteins regulate the opening of mitochondrial permeability transition pores, which can be controlled by Akt signaling [16]. Strongly reduced expression levels of Bcl-2, P-Akt, and P-mTOR, and increased expressions levels of Bax, Bad, cleaved-caspase-3 and -8 were observed in 24-h l-Glu-treated HT22 cells (Figure 3). The 3-h Evo pre-treatment significantly reversed these alterations of anti- and pro-apoptotic protein expression (Figure 3). Evo alone showed no significant effects on the expression levels of Bcl-2, Bax, Bad, cleaved-caspase-8 and P-mTOR in normal HT22 cells (Figure S1E,F). Interestingly, 24-h Evo exposure reduced the expression levels of cleaved-caspase-3 (Figure S1E) and increased the expression levels of P-Akt in the HT22 cells (Figure S1F).

### 2.3. Evo Improved AD-Like Behavior in d-gal and AlCl_3_-Induced AD Mice

**Figure 4:**
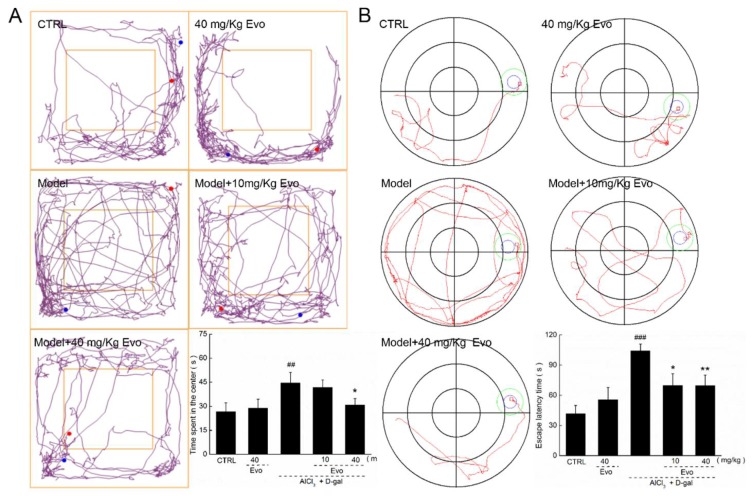
Evo improved the behavioral performances of AD mice. Compared with non-treated AD mice, 42-day Evo administration (**A**) decreased the time mice spent in the central area in the open field test and (**B**) reduced the escape latency time in the Morris water maze test. Data are expressed as mean ± S.E.M. (*n* = 18). ## *p* < 0.01 and ### *p* < 0.001 vs. healthy mice (CTRL), * *p* < 0.05 and ** *p* < 0.01 vs. AD mice (model).

Based on our previous studies [11], an AlCl_3_ and d-gal established AD mouse model was applied in this experiment. The application of 10 and 40 mg/kg of Evo had no significant effect on the bodyweight of AD mice during the 42-day administration period (Table S1). An open field test was used to evaluate the experimental animals’ autonomous exploration behavior and tension in new environments [17,18]. Chaotic movements without purpose were noted in AD mice during the open field test (*p* < 0.01; Figure 4A). Comparatively, 42-day Evo administration, especially at 40 mg/kg, significantly reduced these aimless motions and the time spent in the central area (*p* < 0.05; Figure 4A). The purple lines mean the movement track of mice. 

The Morris water maze was used to detect the long-term spatial learning and memory capability of mice [18,19]. AD mice showed chaotic movements and spent more time finding the hidden platform (*p* < 0.001; Figure 4B) than Evo-treated mice (*p* < 0.05; Figure 4B). The red lines mean the movement track of mice, and blue and green circles mean the platforms. 

All of these data suggest that Evo strongly improved the cognitive abilities of AD mice. 

### 2.4. The Effect of Evo on the Levels of Aβ42 and Aβ40 in Serum and Cerebral Cortex of AD Mice

**Figure 5:**
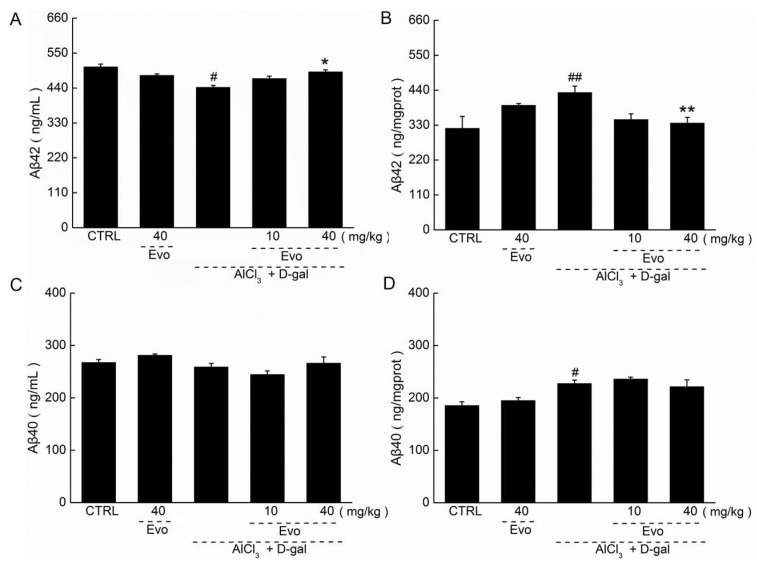
Forty-two-day Evo administration (**A**) enhanced the levels of Aβ42 in serum and (**B**) reduced the levels of Aβ42 in the cerebral cortices, but failed to influence the levels of Aβ40 in the (**C**) serum and (**D**) cerebral cortices of AD mice. Data are expressed as mean ± S.E.M. (*n* = 9). # *p* < 0.05 and ## *p* <0.01 vs. healthy mice (CTRL), * *p* < 0.05, and ** *p* < 0.01 vs. AD mice (model).

The increased level of Aβ42 in the cerebral cortex is a striking feature of AD [2]. Low levels of Aβ42 in serum (*p* < 0.01; Figure 5A) and high levels of Aβ42 in the cerebral cortex (*p* < 0.01; Figure 5B) were found in AD mice. Forty-two-day Evo administration enhanced the Aβ42 level in serum by 11.1% (*p* < 0.05; Figure 5A) and reduced the Aβ42 level in the cerebral cortex by 22.2% (*p* < 0.01; Figure 5B). However, Evo showed no significant effects on the level of Aβ40 in serum (Figure 5C) and cerebral cortex of AD mice (Figure 5D).

### 2.5. The Effect of Evo on the Pathology of the Brain, Spleen, and Kidney in AD Mice

**Figure 6:**
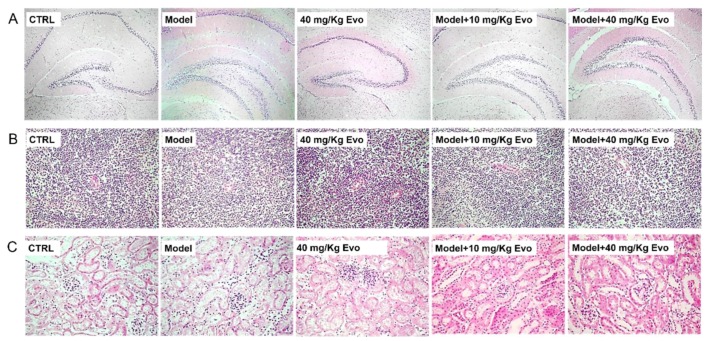
No significant pathologic changes were noted in (**A**) the hippocampus (magnification 40×); (**B**) spleen (magnification 200×); and (**C**) kidney (magnification 200×) among all experimental groups (*n* = 9).

Among all groups, no significant changes were observed in the brain, spleen, and kidney detected via hematoxylin-eosin staining (Figure 6), which suggested that using Evo in mice was safe.

### 2.6. Evo Regulated the Levels of Cholinergic Neurotransmitters in AD Mice

The central cholinergic system is responsible for the dysfunction of memory and cognition, which leads to the development of AD [7]. In AD patients, the remarkable reduction of acetylcholine (Ach) and ChAT and the increment of acetylcholine esterase (AchE) are the most characteristic neurobiological changes [6]. Hypo-levels of Ach and ChAT and hyper-levels of AchE were observed in the serum, cerebral cortex, and hypothalamus of AD mice compared with untreated healthy mice (*p* < 0.05; Table 1). Comparatively, six-week Evo administration significantly reduced the level of AchE (*p* < 0.05; Table 1) and enhanced the levels of Ach and ChAT (*p* < 0.05; Table 1) in the serum, cerebral cortex, and hypothalamus of mice with AD-like behavior.

### 2.7. Evo Displayed Anti-Oxidative Effects in AD Mice

The neuroprotective properties of Evo via the modulation of oxidative stress were detected in the present experiments by determining the levels of ROS, superoxide dismutase (SOD), and glutathione peroxidase (GSH-Px). In AD mice, high levels of ROS and low levels of SOD and glutathione peroxidase (GSH-Px) were noted in the serum, hypothalamus, and cerebral cortex (*p* < 0.05; Table 2), which were significantly reversed by Evo administration (*p* < 0.05; Table 2).

## 3. Discussion

AD, the most common form of dementia in older people, has become a major global health concern with huge implications for individuals and society [20]. Natural compounds have been widely used in the treatment of dementia over thousands of years [21]. In the present study, we successfully confirmed the protective effect of Evo in HT22 cells, as suggested by the enhanced cell viability, reduced cell apoptosis rate, and reversed MMP dissipation and ROS overproduction. Mitochondria are crucial for the maintenance of neuronal integrity and responsiveness [22], and they represent the major source of intracellular ROS [23]. ROS, generated in the electron transport chain, may cause mitochondrial respiratory chain dysfunction, mitochondrial swelling, and MMP dissipation [24]. We found that Evo not only reversed the dissipation of MMP and the over-accumulation of ROS, but also raised the expressions of P-Akt and P-mTOR. The Akt/mTOR signaling pathway is a central axis in the regulation of cellular processes, particularly in neurological diseases [25]. It can negatively regulate the function or expression of several Bcl-2 homology domain 3 (BH3)-only proteins, for instance, it directly phosphorylates and inhibits Bad and Bax [26]. Consequently, an imbalance of Bax and Bcl-2 proteins leads to the dissipation of MMP, which triggers caspase-3 activation and results in cell apoptosis [27]. The neuroprotective effects of Evo on l-Glu-damaged HT22 cells is at least partially related to its improvement of mitochondrial function via the regulation of Akt/mTOR signaling and its downstream proteins.

Behavioral tests are invaluable to researchers attempting to answer questions regarding the mechanisms of behavior. The open field test allows important conventional and ethological parameters to be collected and analyzed [28,29]. In this experiment, we used the routes taken by mice walking back and forth in the open field test to measure their anxiety state, and used the Morris water maze to measure their spatial navigation and memory performance, which helped to confirm the protective effects of Evo in improving AD-like behavior in mice.

In our experiments, d-gal and AlCl_3_ were combined to develop the AD mouse model. Chronic dietary administration of AlCl_3_ can increase Aβ42 levels and accelerate plaque deposition [30]. d-gal, a reducing sugar, induces cognitive impairment and increases oxidative stress [31,32] and damage to cholinergic neurons [33]. Oxidative stress is highly linked to AD pathogenesis [34], especially the initiation of pathological processes [35]. The two key biochemical features of AD are intracellular neurofibrillary tangles (NFTs) and extracellular Aβ plaques. Intracellular NFTs are formed by atypical hyperphosphorylation of tau protein, and ultimately lead to apoptosis and neuronal loss. 42-amino-acid Aβ peptide derived from amyloid precursor protein (APP) is one of the major components of neuritic plaques [2]. The preferential deposition of Aβ42 is attributed to the extended form of COOH of the peptide. Aβ42 is less soluble than Aβ40 and more readily aggregates and forms anti-parallel β-pleated sheets. Aβ42 can also act as seeds to initiate Aβ40 aggregation [2]. Protein oxidation caused by ROS leads to polymerization, thereby forming protein aggregation, such as Aβ deposition [36]. Consequently, the aggregated Aβ reduces mitochondrial respiration in neurons and astrocytes, and induces mitochondrial dysfunction and energy failure [37]. Evo not only suppressed the deposition of Aβ42 in the brains of mice but also regulated the abnormal alternations of oxidative and anti-oxidative factors. SOD is regarded as the first line of the antioxidant defense system against ROS generated in vivo under oxidative stress [14]. Our data suggest that Evo improved AD-like behavior, and the deposition of Aβ42 in brains may be mainly related to its modulation of oxidative stress.

Oxidative stress is responsible for disruption of the cholinergic nervous system, which is thought to be involved in the cognitive impairment associated with AD [38,39]. The aggregated Aβ42 results in the degeneration of cholinergic neurons in the hippocampus and the nucleus basalis, which leads to cholinergic defects and the synapse loss in the AD brain [40]. A decrease in the Ach concentration in the cholinergic synaptic cleft is observed in the AD brain and Aβ negatively regulates the synthesis and release of Ach [41]. In addition, Ach is dynamically controlled by the terminating enzyme AchE and the synthesizing enzyme ChAT [42]. The suppression of AchE activity helps to correct the Ach deficiency [43]. The activity of ChAT, the most specific indicator of the functional state of cholinergic neurons, is decreased in the neocortex of AD patients [44]. Evo significantly ameliorated the abnormal concentration of Ach, AchE, and ChAT in serum and brains, confirming the important role of the cholinergic system regulated by oxidative stress during Evo-mediated neuroprotection in d-gal and AlCl_3_-developed AD mice.

The hypothalamus is the master coordinator of a myriad of homeostatic functions essential for life, such as growth, reproduction, sleep, metabolism, and autonomic homeostasis [45]. ROS overproduction, amyloid plaques, and neurofibrillary tangles in the hypothalamus are candidates for inducing the sympatho-excitation that causes nervous system disease, and the hypothalamus is a target of AD pathology, as are the cortex and hippocampus [46]. Although in the present study we investigated the levels of oxidative stress factors and cholinergic neurotransmitters in the hypothalamus, the relationship between them within Evo-mediated protection against AD still needs further study. 

In conclusion, Evo exerts a protective effect against AD by modulating oxidative stress and reducing the apoptosis rate in l-Glu-induced HT22 cells and experimental AD mice, suggesting its potential benefit for AD patients.

## 4. Materials and Methods

### 4.1. Chemicals and Antibodies

The monomer of Evodiamine (Evo, 518-17-2) and the enzyme-linked immunosorbent assay (ELISA) kit used for measuring the level of Aβ42, Aβ40, Ach, AchE, ChAT, ROS, SOD, and GSH-PX were purchased from Shanghai Yuanye Biological Technology Co., Ltd. (Shanghai, China). Dulbecco’s Modified Eagle Medium (DMEM), 10% fetal bovine serum (FBS), penicillin, and streptomycin were purchased from Invitrogen (Carlsbad, CA, USA). l-Glu, 3-(4, 5-dimethylthiazol-2-yl)-2, 5-diphenyltetrazolium bromide (MTT), dimethyl sulfoxide (DMSO), 2′,7′-dichlorofluorescein diacetate (DCFH-DA), radio immunoprecipitation assay (RIPA) buffer, cocktail, and phenylmethanesulfonyl fluoride (PMSF) were purchased from Sigma-Aldrich (St. Louis, MO, USA). 5,5′,6,6′-tetrachloro-1,1′,3,3′-tetraethylbenzimidazol-ylcarbocyanine iodide (JC-1) was purchased from Calbiochem (San Diego, CA, USA). Polyvinylidene difluoride membranes (0.45 μm) and enhanced chemiluminescence (ECL) kits were purchased from Merck Millipore (Billerica, MA, USA). Primary antibodies Bax (21kd, ab32503), Bad (23kd, ab32445), cleaved-caspase-3 (32kd, ab2302), cleaved-caspase-8 (57kd, ab181580), P-Akt (phosphor S473) (60kd, S473, ab18206), T-Akt (60kd, ab106693), P-mTOR (phosphor, S2448) (289kd, S2448, ab109268), T-mTOR (289kd, ab83495), and glyceraldehyde-3-phosphate dehydrogenase (GAPDH) (36kd, ab181602) were purchased from Abcam (Cambridge, MA, USA). Bcl-2 (bsm-33047M) was purchased from Bioss (Beijing, China).

### 4.2. Cell Culture

The mouse hippocampal neuronal cell lines (HT22; BNCC; 337709) were cultured in DMEM, supplemented with 10% heat-inactivated FBS, 100 μg/mL streptomycin, and 100 units/mL penicillin and incubated in a 5% CO_2_ humidified incubator at 37 °C.

### 4.3. Cell Viability Assay

HT22 cells were plated (10,000 cells/well) in 96-well plates, and pretreated with Evo at doses of 5, 10, 20, and 40 μM for 3 h and then co-exposed to 25 mM l-Glu for another 24 h at 37 °C. In a separate experiment, HT22 cells were exposed to 20 μM Evo alone for 24 h at 37 °C. The culture medium was removed and incubated with MTT (0.5 mg/mL) at 37 °C for 4 h. After adding DMSO, the optical density at a wavelength of 540 nm was read by a Synergy^TM4^ Microplate Reader (BioTek Instruments, Winooski, VT, USA).

### 4.4. Cell Apoptosis Assay

HT22 cells were pretreated with 5 and 20 μM Evo for 3 h, and then co-exposed with 25 mM l-Glu for another 24 h. In a separate experiment, HT22 cells were treated with 20 μM Evo alone for 24 h at 37 °C. The fluorescence intensity of cells incubated with propidium iodide (PI) and annexin V (AV) for 20 min at room temperature in darkness was measured using a Muse^TM^ Cell Analyzer from Millipore (Billerica, MA, USA) according to the manufacturer’s instructions.

### 4.5. Measurement of MMP and Intracellular ROS Levels

MMP was measured using JC-1 staining. HT22 cells were pretreated with 5 and 20 μM Evo for 3 h and then co-exposed with 25 mM l-Glu for another 12 h. In a separate experiment, HT22 cells were incubated with 20 μM Evo alone for 12 h at 37 °C. The cells were then exposed to JC-1 for 15 min at 37 °C. The green/red fluorescence intensity was analyzed using a Nikon Eclipse TE 2000-S fluorescence microscope (Nikon, Tokyo, Japan). 

To evaluate the levels of intracellular ROS, the treated cells were stained with 10 μM DCFH-DA for 15 min at 37 °C in the dark. The green fluorescence intensity was detected by a Nikon Eclipse TE 2000-S fluorescence microscope (Nikon, Japan).

### 4.6. Western Blot

HT22 cells were pre-treated with Evo at 25 and 100 μM for 3 h and co-incubated with 25 mM l-Glu for 24 h. In a separate experiment, HT22 cells were exposed to 20 μM Evo alone for 24 h. The incubated cells were lysed with RIPA buffer containing 1% protease inhibitor cocktail and 2% PMSF. Lysed cell proteins were quantified using the BCA protein assay kit. Forty micrograms of lysates were resolved by 12% SDS-PAGE, and transferred to polyvinylidene difluoride membranes (0.45 μm, Merck Millipore, Billerica, MA, USA). The membranes were blocked with 5% bovine serum albumin for 2 h at 4 °C and then incubated with primary antibodies, including Bax, Bcl-2, Bad, cleaved-caspase-3 and -8, P-Akt, T-Akt, P-mTOR, T-mTOR, and GAPDH, at 4 °C overnight, followed by incubation with horseradish peroxidase-conjugated secondary antibody at 4 °C for 4 h. An ECL kit and imaging system (Bio Spectrum 600, UVP company, Upland, CA, USA) were applied to detect blots, and the bands were quantified by ImageJ software (National Institutes of Health, Bethesda, MD, USA).

### 4.7. The Development of AD Mouse Model and Agent Administration Process

The experiment was conducted with the approval of the Institution Animal Ethics Committee of Jilin University (License No.: 20160518) at 18 May 2016. Ninety BALB/c mice (6–8 weeks; 18–20 g) were purchased from Norman Bethune University of Medical Science, Jilin University, Changchun, Jilin, China (SCXK (LIAO)-2015-0001). Mice were randomly divided into five groups (*n* = 18/group), placed in a room at a temperature of 23 ± 2 °C, and a humidity of 40–60% with the circadian rhythm of light, and given free water and food.

**Figure 7:**
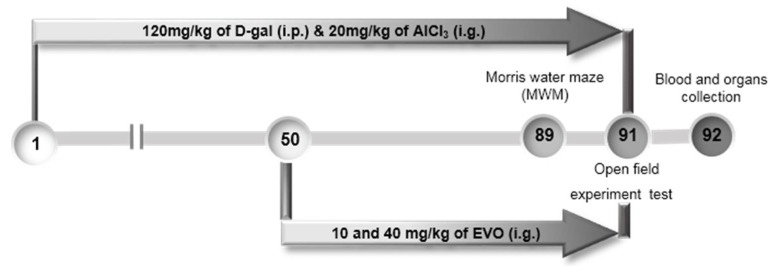
Schematic of experiments. The AD mouse model was established by administering AlCl_3_ (20 mg/kg, i.g.) and d-gal (120 mg/kg i.p.) daily for 91 days. From the 50th day, the mice were given Evo at doses of 10 and 40 mg/kg daily for 42 days. Behavioral tests were performed starting from the 89th day.

Fifty-four mice were injected with 120 mg/kg of d-gal subcutaneously and intragastrically administrated with 20 mg/kg of AlCl_3_ once daily for 91 days. From the 50th day, Evo-treated mice were given 10 and 40 mg/kg of Evo (*n* = 18/group), and model mice (*n* = 18) were fed with double distilled water (D.D.) water each day for 42 days. The control mice (*n* = 18) were intraperitoneally and intragastrically treated with normal saline throughout the whole experiment (91 days). Mice treated Evo alone (*n* = 18) were intraperitoneally and intragastrically administrated with saline throughout the whole experiment and intragastrically treated with 40 mg/kg of Evo from the 50th day. After the agent administration phase, the mice were evaluated by a series of behavioral tests, and blood was then collected from the tails of mice under anesthesia with 10% chloral hydrate, and the brain and hypothalamus were removed and homogenized in saline. The entire brain hemisphere, the kidney, and the spleen were collected and immersed in 4% formalin for pathologic analysis (Figure 7).

### 4.8. Behavioral Tests

#### 4.8.1. Open Field Experiment Test

This experiment was used to observe the spontaneous exploration motor activity of mice after the administration of agents. Each mouse was placed in one of the four corner squares facing the wall and allowed to freely explore the environment for 5 min. A quiet environment was ensured throughout the experiment. The instrument recorded the latency time for the mouse to walk out of the central and surrounding areas. Each mouse was wiped off after completing the experiment to avoid leaving odors and dirt that could interfere with the results of the experiment.

#### 4.8.2. Morris Water Maze Test

The Morris water maze was used to assess brain function associated with spatial learning and memory via the MT-200 Water Labyrinth Video Tracking Analysis System (S7200, Chengdu Techman Software Co., Ltd., Chengdu, China), with a circular pool filled to a depth of 10 cm with water (24–26 °C) containing 1 L of milk, which was 2 cm higher than the platform. The training started from the 85th day. The formal test started after 5 days of training. The latency from the immersion of the mouse in the pool to its escape onto the hidden platform within 120 s was recorded.

### 4.9. Enzyme-Linked Immunosorbent Assay

The levels of Ach, AchE, ChAT, ROS, SOD, and GSH-Px in the serum, hypothalamus, and cerebral cortex, and the levels of Aβ42 and Aβ40 in the serum and cerebral cortex lysates were measured by ELISA according to the procedures provided by the related assay kits.

### 4.10. Histological Examination

The fixed whole brain hemisphere, the kidney, and the spleen were washed, dehydrated with alcohol, and finally embedded in paraffin. The whole hemisphere, the kidney, and the spleen were cut into standard sections and stained with hematoxylin–eosin for histological examination, similar to our previous study [47].

### 4.11. Statistical Analysis

All data are presented as the mean ± S.E.M. One-way analysis of variance (ANOVA) was used to analyze the significance of the statistical results. Differences were considered significant when *p* < 0.05.

## Figures and Tables

**Table 1:** Evo regulated the levels of Ach, AchE, and ChAT in the serum, hypothalamus, and cerebral cortex.

Items	Neurotransmitters	CTRL	Evo (40 mg/kg)	AlCl_3_ + d-gal		
				Model	Evo (mg/kg)	
					10	40
Serum	Ach (μg/mL)	886.8 ± 22.6	895.0 ± 31.4	773.3 ± 14.2 ##	845.0 ± 28.2 **	916.7 ± 26.7 **
	AchE (nmol/L)	146.8 ± 4.0	138.7 ± 10.0	174.7 ± 7.8 ##	132.0 ± 4.8 **	152.7 ± 4.2 *
	ChAT(pmol/L)	275.7 ± 11.0	259.3 ± 6.2	231.4 ± 10.7 #	260.2 ± 7.2 *	264.3 ± 4.3 *
Hypothalamus	Ach (μg/mgprot)	271.2 ± 27.2	249.2 ± 22.5	176.5 ± 18.5 ##	209.3 ± 14.5	258.6 ± 13.1 **
	AchE (nmol/gprot)	56.7 ± 4.2	60.3 ± 4.4	72.4 ± 4.4 ##	66.1 ± 5.3	58.7 ± 1.8 *
	ChAT (pmol/gprot)	126.3 ± 12.8	123.3 ± 14.5	75.8 ± 6.4 ###	87.0 ± 7.7	145.9 ± 16.7 ***
Cerebral Cortex	Ach (μg/mgprot)	765.9 ± 23.1	748.7 ± 23.8	546.3 ± 10.7 #	615.2 ± 17.5 *	626.4 ± 21.5 *
	AchE (nmol/gprot)	58.5 ± 6.8	66.5 ± 5.0	103.5 ± 5.1 ###	84.9 ± 2.9 **	73.6 ± 3.2 ***
	ChAT (pmol/gprot)	240.0 ± 15.8	232.5 ± 4.3	165.1 ± 3.1 #	243.0 ± 14.0 **	212.2 ± 12.8 *

Data expressed as mean ± S.E.M. (*n* = 9). # *p* < 0.05, ## *p* < 0.01 and ### *p* < 0.001 vs. healthy mice (CTRL), * *p* < 0.05, ** *p* < 0.01 and *** *p* < 0.001 vs. AD mice (Model).

**Table 2:** Evo regulated the levels of ROS, SOD, and GSh-PX in the serum, hypothalamus, and cerebral cortex.

Items	Factors	CTRL	Evo (40 mg/kg)	AlCl_3_ + d-gal		
				Model	Evo (mg/kg)	
					10	40
Serum	ROS (U/mL)	458.8 ± 6.0	465.5 ± 6.3	535.2 ± 6.2 #	515.6 ± 13.3	482.5 ± 16.5 *
	SOD (U/mL)	322.1 ± 3.7	322.9 ± 7.3	282.5 ± 18.3 #	310.8 ± 7.2 *	330.8 ± 18.7 *
	GSH-Px (U/mL)	748.5 ± 25.2	792.5 ± 55.2	655.4 ± 23.0 #	711.7 ± 6.7	765.0 ± 10.2 *
Hypothalamus	ROS (U/mgprot)	123.5 ± 9.9	103.8 ± 8.4	160.8 ± 10.5 ##	137.6 ± 10.3	122.4 ± 6.0 **
	SOD (U/mgprot)	123.2 ± 12.6	112.6 ± 14.7	70.8 ± 7.5 ###	98.4 ± 3.7 *	116.2 ± 15.5 **
	GSH-Px (U/mgprot)	198.5 ± 18.7	191.9 ± 25.1	136.0 ± 13.6 ##	199.5 ± 11.9 **	236.4 ± 26.3 **
Cerebral Cortex	ROS(U/mgprot)	208.2 ± 15.8	220.1 ± 14.2	375.7 ± 20.2 ###	363.1 ± 15.9	313.4 ± 13.9 *
	SOD(U/mgprot)	164.7 ± 15.2	189.6 ± 17.9	121.4 ± 12.2 #	164.1 ± 4.7 *	183.9 ± 6.0 *
	GSH-Px (U/mgprot)	335.7 ± 20.6	349.3 ± 9.2	265.6 ± 3.5 #	321.7 ± 11.3 *	339.3 ± 21. 1*

Data expressed as mean ± S.E.M. (*n* = 9). # *p* < 0.05, ## *p* < 0.01, and ### *p* < 0.001 vs. healthy mice (CTRL), * *p* < 0.05, ** *p* < 0.01, and *** *p* < 0.001 vs. AD mice (Model).

## Supplementary Materials

The following are available online at http://www.mdpi.com/1422-0067/19/5/1527/s1.

## Abbreviations

Ach	acetylcholine
AchE	acetylcholine esterase
AD	Alzheimer’s disease
Akt	protein kinase B
AlCl_3_	aluminum trichloride
ANOVA	one-way analysis of variance
Aβ	amyloid beta
Bcl-2	B-cell lymphoma-2
ChAT	choline acetyltransferase
d-gal	d-galactose
Evo	Evodiamine
ELISA	enzyme-linked immunosorbent assay
GAPDH	glyceraldehyde-3-phosphate dehydrogenase
GSH-Px	glutathione peroxidase
H&E	hematoxylin–eosin staining
l-Glu	l-glutamic acid
MAPK	mitogen-activated protein kinase
MMP	mitochondrial membrane potential
mTOR	mammalian target of rapamycin
MWM	Morris water maze test
ROS	reactive oxygen species
SOD	superoxide dismutase
