# Supplementary material for: Pharmacological Basis for the Use of Evodiamine in Alzheimer’s Disease: Antioxidation and Antiapoptosis
Source: Int J Mol Sci. 2018 May 21;19(5):1527. doi: 10.3390/ijms19051527 (PMC5983845; doi:10.3390/ijms19051527)
Supplement: Supplementary file 1 [file ijms-19-01527-s001.pdf]

Supplementary data

Table S1. The effects of Evo on bodyweights in Alzheimer's disease mice

	Doses	91-day treatment						
		0	21	35	49	63	77	91
CTRL	--	20.8±0.5	25.8±0.3	26.7±0.3	28.0±0.5	28.8±0.5	29.2±0.4	29.3±0.4
Evo	40mg/kg	21.2±0.3	25.7±0.2	24.8±0.5	28.3±0.3	29.7±0.2	30.3±0.1	30.0±0.2
$AlCl_3$ + D-gal	--	21.3±0.3	23.5±0.2	23.0±0.5	26.5±0.4	27.7±0.2	28.7±0.2	27.7±0.4
$AlCl_3$ + D-gal +Evo	10mg/kg	20.1±0.5	24.3±0.3	24.4±0.4	27.2±0.3	27.9±0.3	28.2±0.3	28.4±0.2
$AlCl_3$ + D-gal +Evo	40mg/kg	19.9±0.5	24.9±0.3	24.5±0.5	27.4±0.4	27.4±0.3	27.8±0.3	28.2±0.3

Data are expressed as mean ± S.E.D. (n=18) and analyzed by using a one-way ANOVA.

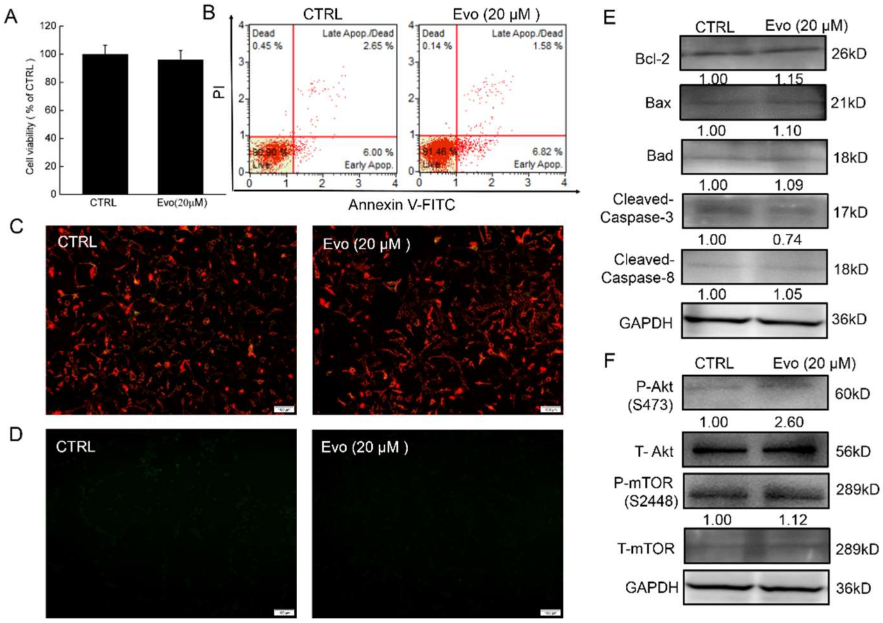

Figure S1. 24-h incubation with 20 μM of Evo had no significant effects on (A) cell viability, (B) apoptosis rate, (C) the dissipation of MMP and (D) the over-accumulation of ROS in HT22 cells (n=10). (E and F) Evo reduced the expression levels of cleaved-caspase-3, increased the expression levels of P-Akt, but showed no significant effect on the expression levels of Bcl-2, Bax, Bad, P-mTOR and cleaved-caspase-8. Quantification data were normalized by (E) GAPDH and (F) corresponding total proteins, and the average fold increase in band intensity compared with the CTRL group are marked respectively (n=6).
